# Native Aortic Valve Disease Progression and Bioprosthetic Valve Degeneration in Patients With Transcatheter Aortic Valve Implantation

**DOI:** 10.1161/CIRCULATIONAHA.121.056891

**Published:** 2021-08-29

**Authors:** Jacek Kwiecinski, Evangelos Tzolos, Timothy R.G. Cartlidge, Alexander Fletcher, Mhairi K. Doris, Rong Bing, Jason M. Tarkin, Michael A. Seidman, Gaurav S. Gulsin, Nicholas L. Cruden, Anna K. Barton, Neal G. Uren, Michelle C. Williams, Edwin J.R. van Beek, Jonathon Leipsic, Damini Dey, Raj R. Makkar, Piotr J. Slomka, James H.F. Rudd, David E. Newby, Stephanie L. Sellers, Daniel S. Berman, Marc R. Dweck

**Affiliations:** Department of Interventional Cardiology and Angiology, Institute of Cardiology, Warsaw, Poland (J.K.).; Centre for Cardiovascular Science (E.T., T.R.G.C., A.F., M.K.D., R.B., N.L.C., A.K.B., N.G.U., M.C.W., E.J.R.v.B., D.E.N., M.R.D.), University of Edinburgh, UK.; Edinburgh Imaging, facility QMRI (E.J.R.v.B.), University of Edinburgh, UK.; Department of Radiology, Centre for Cardiovascular Innovation, & Centre for Heart Lung Innovation, University of British Columbia & St. Paul’s Hospital, Canada (J.Z.S., G.S.G., J.L., S.K.S.).; Department of Imaging (Division of Nuclear Medicine), Medicine, and Biomedical Sciences, Cedars-Sinai Medical Center, Los Angeles, CA (D.D., R.R.M., P.J.S., D.S.B.).; Division of Cardiovascular Medicine, University of Cambridge, UK (J.M.T., J.H.F.R.).

**Keywords:** aortic valve, positron emission tomography computed tomography, 18F-sodium fluoride, transcatheter aortic valve implantation

## Abstract

**Background:**

Major uncertainties remain regarding disease activity within the retained native aortic valve, and regarding bioprosthetic valve durability, after transcatheter aortic valve implantation (TAVI). We aimed to assess native aortic valve disease activity and bioprosthetic valve durability in patients with TAVI in comparison with subjects with bioprosthetic surgical aortic valve replacement (SAVR).

**Methods:**

In a multicenter cross-sectional observational cohort study, patients with TAVI or bioprosthetic SAVR underwent baseline echocardiography, computed tomography angiography, and ^18^F-sodium fluoride (^18^F-NaF) positron emission tomography. Participants (n=47) were imaged once with ^18^F-NaF positron emission tomography/computed tomography either at 1 month (n=9, 19%), 2 years (n=22, 47%), or 5 years (16, 34%) after valve implantation. Patients subsequently underwent serial echocardiography to assess for changes in valve hemodynamic performance (change in peak aortic velocity) and evidence of structural valve dysfunction. Comparisons were made with matched patients with bioprosthetic SAVR (n=51) who had undergone the same imaging protocol.

**Results:**

In patients with TAVI, native aortic valves demonstrated ^18^F-NaF uptake around the outside of the bioprostheses that showed a modest correlation with the time from TAVI (*r*=0.36, *P*=0.023). ^18^F-NaF uptake in the bioprosthetic leaflets was comparable between the SAVR and TAVI groups (target-to-background ratio, 1.3 [1.2–1.7] versus 1.3 [1.2–1.5], respectively; *P*=0.27). The frequencies of imaging evidence of bioprosthetic valve degeneration at baseline were similar on echocardiography (6% versus 8%, respectively; *P*=0.78), computed tomography (15% versus 14%, respectively; *P*=0.87), and positron emission tomography (15% versus 29%, respectively; *P*=0.09). Baseline ^18^F-NaF uptake was associated with a subsequent change in peak aortic velocity for both TAVI (*r*=0.7, *P*<0.001) and SAVR (*r*=0.7, *P*<0.001). On multivariable analysis, ^18^F-NaF uptake was the only predictor of peak velocity progression (*P*<0.001).

**Conclusions:**

In patients with TAVI, native aortic valves demonstrate evidence of ongoing active disease. Across imaging modalities, TAVI degeneration is of similar magnitude to bioprosthetic SAVR, suggesting comparable midterm durability.

**Registration:**

URL: https://www.clinicaltrials.gov; Unique identifier: NCT02304276.

## Abstract 2

Supplemental Digital Content is available in the text.

**Clinical Perspective**

**What Is New?**

- After transcatheter aortic valve implantation, native aortic valves demonstrate evidence of ongoing disease activity, suggesting that aortic stenosis is an active disease process that is independent of motion and mechanical injury.
- ^18^F-sodium fluoride positron emission tomography identifies subclinical bioprosthetic degeneration of transcatheter aortic valves, providing prediction of subsequent valvular dysfunction and highlighting patients at risk of valve failure.
- Across 3 complementary and distinct imaging modalities, bioprosthetic degeneration of transcatheter aortic valves appears to be of a magnitude similar to bioprosthetic surgical aortic valve replacement, suggesting comparable midterm durability.

**What Are the Clinical Implications?**

- ^18^F-sodium fluoride positron emission tomography holds promise in the detection of bioprosthetic aortic valve degeneration and prediction of bioprosthesis failure.

Transcatheter aortic valve implantation (TAVI) has revolutionized intervention options in aortic valve stenosis.^1–4^ Although the terms TAVI and transcatheter aortic valve replacement are widely used interchangeably, transcatheter aortic valve replacement is a misnomer because the native aortic valve is not replaced but rather displaced and splinted against the wall of the aorta at the time of bioprosthetic valve insertion. As a consequence, the native aortic valve is rendered immobile. It has been previously suggested that the impact of repeated valve closure and trauma is fundamental to aortic stenosis.^5^ Therefore, patients with TAVI present a unique opportunity to investigate the pathophysiology of aortic stenosis in the absence of the ongoing cyclic mechanical trauma of valve closure. Is aortic stenosis simply a disease of wear-and-tear or is it an active regulated pathobiological process that continues despite valve immobilization?

TAVI is rapidly gaining popularity as a treatment option in younger low-risk populations.^2–4^ With its more widespread use, questions regarding valve durability become increasingly important.^6^ All bioprosthetic valves are susceptible to degeneration, driven by processes similar to native aortic valve stenosis. Active calcification appears to be the final common pathway of such degeneration leading to bioprosthetic valve stenosis, leaflet tears, and valvular regurgitation.^7,8^ Although transcatheter bioprostheses are similar in structure to surgical valves, it has been suggested that the increased effective orifice area of TAVI will result in improved longevity. However, others have proposed that crimping of TAVI bioprostheses coupled with incomplete asymmetrical frame expansion and suboptimal leaflet coaptation may lead to accelerated structural valve deterioration.^9^ Although long-term hemodynamic valve data are lacking, there is interest in comparing earlier noninvasive markers of valve durability in patients with TAVI and those with bioprosthetic surgical aortic valve replacement (SAVR).

We have demonstrated that ^18^F-sodium fluoride (^18^F-NaF) positron emission tomography (PET) provides a marker of calcification activity and vascular injury across a range of cardiovascular conditions.^10–15^ In native aortic valve stenosis, ^18^F-NaF uptake can assess valve calcification activity, providing important pathophysiological insights, a measure of disease severity, and act as a predictor of subsequent disease progression and clinical events.^10,11^ In bioprosthetic SAVR, ^18^F-NaF PET uptake is an early and sensitive marker of leaflet degeneration, providing powerful prediction of subsequent valve dysfunction and valve failure.^12^

In the present study, we sought to investigate whether the retained native aortic valves in patients undergoing TAVI demonstrate evidence of ongoing disease progression. In addition, because the long-term durability of transcatheter aortic valves is yet to be established, we aimed to establish whether bioprosthetic valve durability or degeneration was appreciably different between patients with TAVI or SAVR at midterm follow-up.

## Methods

### Study Design and Patient Population

**Figure 1.**
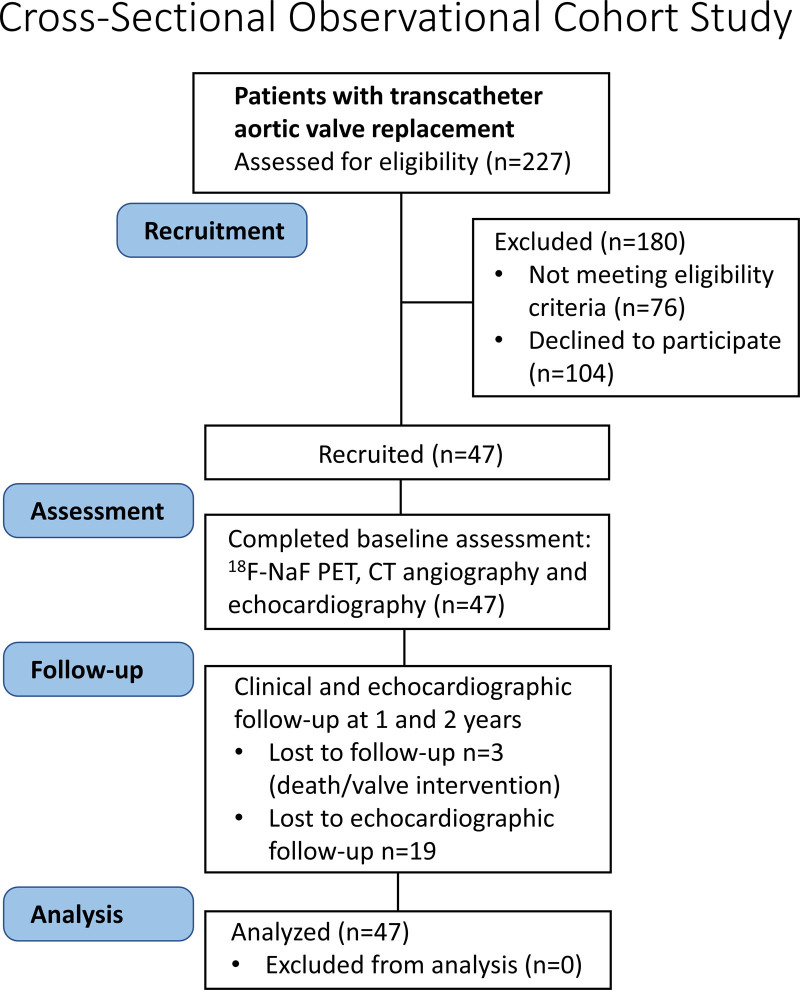
**CONSORT flow diagram of study recruitment, allocation (assessments), follow-up, and analysis.** CT indicates computed tomography; and ^18^F-NaF PET, ^18^F-sodium fluoride positron emission tomography.

Patients with aortic stenosis who had undergone previous TAVI (1 month, 2 years, or 5 years before study inclusion) using a balloon-expandable or self-expanding bioprosthesis were prospectively recruited into an observational cross-sectional cohort study at 3 high-volume TAVI centers between September 2016 and November 2019 (Edinburgh Heart Center, Cedars Sinai Medical Center, and Cambridge University Addenbrooke’s Hospital; Figure 1). All participants were under routine clinical follow-up and did not have established clinical evidence of bioprosthetic valve degeneration.^16^ Each patient underwent clinical assessment, echocardiography, hybrid ^18^F-NaF PET, and computed tomography (CT) angiography at baseline with annual repeat echocardiography thereafter (Figure 1). We excluded patients unable to give informed consent, with claustrophobia, allergy to iodinated contrast, liver failure, chronic kidney disease (with estimated glomerular filtration rate <30 mL·min^–1^·1.73 m^–2^), Paget disease, metastatic malignancy, or an inability to tolerate the supine position. Patients with TAVI valves were compared with patients with SAVR valves undergoing the same research protocol (including multimodality imaging protocols, image analysis assessments, and follow-up (URL: http://www.clinicaltrials.gov; Unique identifier: NCT02304276). Patients were recruited prospectively, matching the age of SAVR and TAVI valves (time from valve implantation for aortic stenosis to imaging) in the 2 groups. Baseline and follow-up data from the SAVR cohort in isolation have been reported previously.^12^ The study (URL: http://www.clinicaltrials.gov; Unique identifier: NCT02304276) was conducted in accordance with the Declaration of Helsinki and was approved by National Health Service Scotland Research Ethics Committee (14/SS/1049), the Administration of Radioactive Substances Advisory Committee, and the institutional review boards at all sites. Recruitment was prematurely halted because of the onset of the severe acute respiratory syndrome coronavirus 2 (SARS-CoV-2) pandemic and the potential vulnerability of our target population. In addition, we encountered difficulties in recruiting patients at 5 years after TAVI who were both alive and well enough to undergo study procedures. The data that support the findings of this study are available from the corresponding author on reasonable request.

### Aortic Valve Imaging

#### Echocardiography

Two-dimensional and Doppler echocardiography was performed at baseline and annually thereafter according to American Society of Echocardiography guidelines.^17^ Aortic valve Doppler measurements were routinely assessed from the apex, suprasternal notch, and right sternal edge to measure the peak aortic jet velocity, the mean gradient, and the effective orifice area of the bioprosthesis. Mean values were taken from 3 measurements when subjects were in sinus rhythm and from 5 measurements if they were in atrial fibrillation. Bioprosthetic valve regurgitation was graded as mild, moderate, or severe according to guideline recommendations on the basis of visual appraisal of color Doppler images, measurement of pressure half-time (milliseconds), and assessment for aortic flow reversal in diastole.^17^

#### PET/CT Imaging

All patients underwent ^18^F-NaF PET at baseline on hybrid PET/CT scanners (128-slice Biograph mCT, Siemens Medical Systems, or Discovery 690/710 GE Healthcare) using harmonized imaging protocols, 60 minutes after intravenous administration of 125 MBq of ^18^F-NaF^18^ obtained in 3-dimensional mode in a single 30-minute bed position centered on the valve. Attenuation-correction CT was performed before acquisition of PET data. Last, ECG-gated contrast-enhanced CT angiography was performed on the same scanner with prospective gating in end-expiration. Patients were given β-blockers if resting heart rate was >65 beats/min and in the absence of clinical contraindications. After coregistration with PET, the CT data served for anatomic reference and facilitated PET tracer uptake quantification.^19^

### Imaging Analysis

#### Computed Tomography

Abnormalities on CT angiography were adjudicated using prespecified criteria. Noncalcific leaflet thickening (hypoattenuated leaflet thickening [HALT]) was defined as focal areas of low-attenuation (30–200 Hounsfield Units) leaflet thickening visualized in at least 2 planes typically thickest at its base and thinning to the tips in accordance with consensus guidelines.^20,21^ Pannus was defined as circumferential low-attenuation (noncalcific) material with radial thickness ≥2 mm and encroachment on to the valve cusps.^12^ Leaflet calcification was defined as calcium >500 Hounsfield Units localized to a valve cusp in at least 2 planes and classified according to size as spotty calcification if maximum diameter was <3 mm, or large calcification if maximum diameter was ≥3 mm.^22^

#### Positron Emission Tomography

Reconstructed ECG-gated PET and contrast-enhanced CT images were reoriented, they were coregistered in orthogonal planes, and cardiac motion was corrected with an automatic algorithm preserving counts from all cardiac phases (Data Supplement Methods).^23–26^ With the use of en face images of the bioprosthetic valves, the maximum standard uptake value in the native aortic valve was measured between the perimeter of the TAVI bioprostheses and the aorta. Care was taken to avoid regions of activity originating from the TAVI leaflets and nearby coronary arteries. Tissue-to-background ratio (TBR) values were derived from maximum standard uptake value values corrected for blood-pool activity (mean standard uptake value) measured in the right atrium (1-cm radius 9-mm-high cylinder drawn on axial slices, at the level of the right coronary ostium).

With respect to ^18^F-NaF uptake in the TAVI bioprosthetic valves, PET scans were adjudicated to be abnormal if discernible ^18^F-NaF uptake originating from the valve leaflets was observed on 3 orthogonal planes. We quantified ^18^F-NaF uptake according to a previously proposed methodology where a circular (area 1 cm^2^) region of interest was drawn around the area of maximal uptake originating in the valve cusps.^12,27^ Regions of interest were carefully drawn to avoid any uptake originating from outside the bioprosthetic valve leaflets, in particular, uptake related to surrounding native aortic valve tissue. In subjects with no visible (exceeding blood-pool activity) uptake in the valve leaflets, a 1-cm^2^ circular region of interest was drawn in the center of the valve.^10–12^ Maximum standard uptake values were extracted from these regions of interest and divided by the blood-pool activity measured in the right atrium to calculate the TBR values as described earlier. A similar approach was taken to the analysis of SAVR valves.^12^

### Clinical Follow-Up

Patients were invited to return annually for 2 years for repeat clinical assessment and echocardiography to assess for evidence of deterioration in hemodynamic bioprosthetic performance. In particular, change in peak velocity through the valve, change in mean pressure gradient, and change in the effective orifice area were recorded. Changes in the grade of aortic regurgitation were documented.

Bioprosthetic valve deterioration was determined at baseline and after follow-up and was categorized as: stage 1, a morphological abnormality (detected on echocardiography or CT), including HALT, calcification or pannus, in the absence of hemodynamic changes; stage 2, either moderate valve obstruction, moderate regurgitation or both; and stage 3, either severe valve obstruction or regurgitation.^9,16^

Patients were followed up for clinical events with outcome information obtained from local and national health care record systems that integrate primary and secondary health care records. The primary clinical end point of the study was a composite of bioprosthetic valve failure or repeat TAVI. Categorization of these outcomes was performed blinded to the PET imaging or other study data. Outcome data were collected in September 2020.

#### Ex Vivo Assessment

To elucidate the pathology of aortic stenosis and TAVI degeneration and to validate our in vivo imaging findings, we studied surgically explanted native and bioprosthetic aortic valves obtained from patients with dysfunctional degenerated TAVI in the Cardiovascular Tissue Registry at St. Paul’s Hospital. Ex vivo histological (hematoxylin and eosin; Movat pentachrome), immunohistochemistry (runx2 and osteopontin), and ^18^F-NaF autoradiography assessments^8^ were made on these samples in accordance with the approval of the Research Ethics Board of Providence Health Care (Data Supplement Methods).

### Statistical Analysis

We assessed the distribution of data with the Shapiro-Wilk test. Continuous parametric variables were expressed as mean (SD) and compared using Student *t* tests. Nonparametric data were presented as median (interquartile interval), compared using Mann-Whitney *U* test and log transformed to achieve normality before inclusion in regression models and correlation. Fisher exact test or χ^2^ test was used for analysis of categorical variables. We assessed correlations with the Pearson coefficient. Multivariable linear regression modeling was used to assess the change in echocardiographic measures of bioprosthesis performance, clinical characteristics, and ^18^F-NaF uptake. The multivariable model was constructed with annualized peak velocity change (m/s) as the dependent variable and age, sex, time after aortic valve replacement, presence of HALT, valve TBR, and baseline peak velocity and abnormalities on CT as independent variables, selected on the basis of clinically relevant and plausible mechanisms that may relate to valvular degeneration. Model residuals were checked against fitted values and distributions confirmed with quantile-quantile plots. To assess imaging evidence of bioprosthetic valve degeneration in TAVI or SAVR, we compared the echocardiography, CT, and ^18^F-NaF PET findings in our TAVI population with matched data from a previous study that characterized patients with bioprosthetic SAVR using the same clinical assessments, multimodality imaging protocols, and image analyses.^12^ Receiver operating characteristic analysis was performed to identify the optimum cutoff for TBR to identify patients at increased risk of structural valve degeneration using the Youden J statistic. Statistical analysis was performed with SPSS version 24 (IBM SPSS Statistics for Windows, Version 24.0, IBM Corp), R studio and R software version 4.01 (R Foundation for Statistical Computing). We used R packages: dlpyr, ggplot2, magrittr, QuantPsyc, Forestplot, cutpointr, and ggpubr. A 2-sided *P*<0.05 was considered statistically significant.

## Results

### Study Populations

We recruited 47 patients with TAVI from 3 high-volume centers (81±6 years of age, 79% men) who were compared with 51 patients with SAVR from the same institutions (Table 1). Similar to the SAVR cohort, patients with TAVI were imaged once with ^18^F-NaF PET/CT at either 1 month (n=9, 19%), 2 years (n=22, 47%), or 5 years (16, 34%) after valve implantation. Twenty-five (53%) subjects were implanted with a balloon-expanded bioprosthesis and 22 (47%) received a self-expanding valve.

**Table 1.**
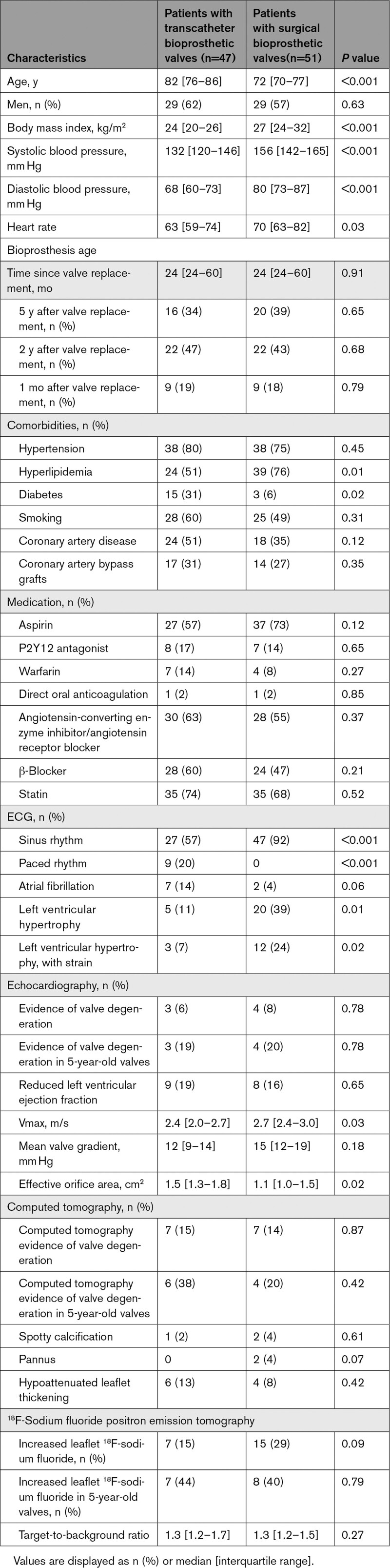
Comparison of Patients After Transcatheter Aortic Valve Implantation Versus Patients After Surgical Aortic Valve Replacement

### Calcification Activity in Native Aortic Valve Tissue

#### Ex Vivo Validation

**Figure 2.**
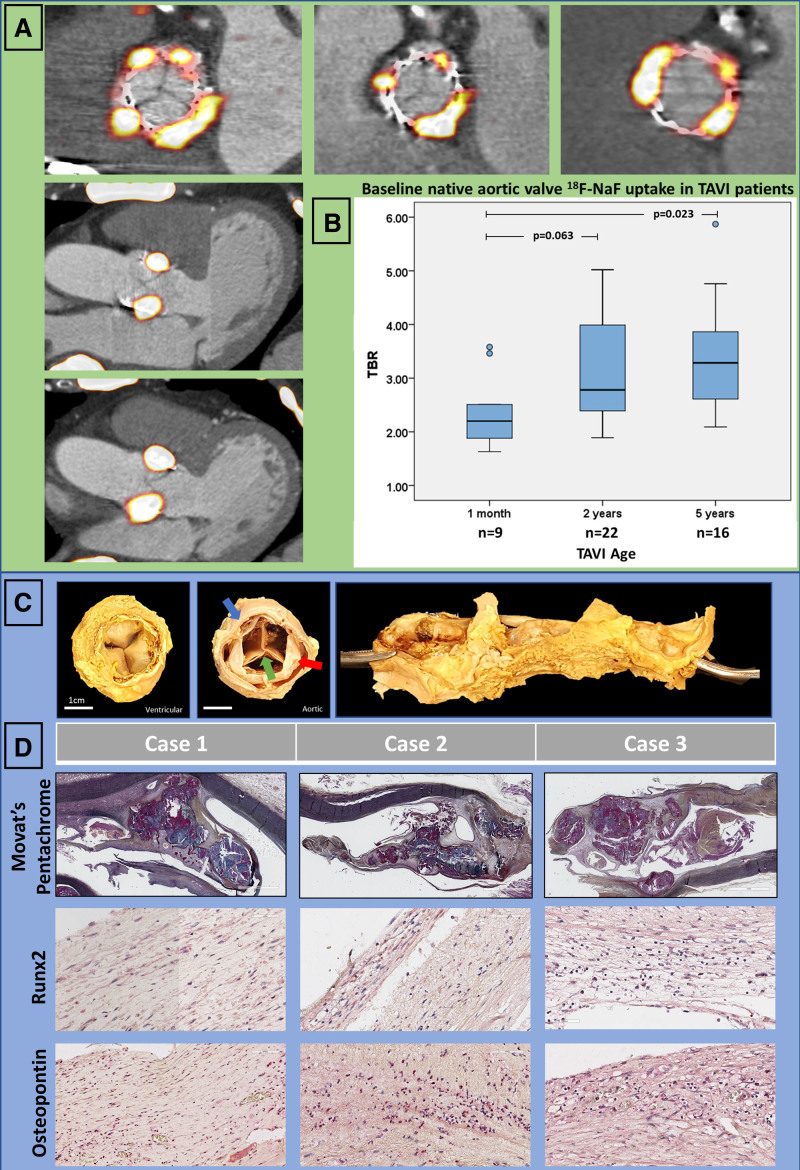
**Baseline assessment with ^18^F-sodium fluoride activity in native aortic valve tissue after transcatheter aortic valve replacement.** **A**, Hybrid ^18^F-sodium fluoride (^18^F-NaF) positron emission tomography and computed tomography en face and long-axis images of native aortic valve tissue uptake. We observed intense tracer activity originating from the native valve tissue around the perimeter of the bioprosthesis in all patients with transcatheter aortic valve implantation (TAVI). **B**, Native aortic valve ^18^F-NaF uptake in patients with TAVI was higher with longer duration because bioprosthesis implantation suggesting increased calcification activity after intervention. **C**, Representative macroscopic images of explanted TAVI valves (green arrow) surrounded by native aortic valve (red arrow) jailed between the bioprostheses and the aortic root (blue arrow): ventricular aspect (**Left**), aortic aspect (**Middle**), and view of the root with native valve tissue cut and opened out along its perimeter (**Right**). **D**, Histology (Movat pentachrome staining) and immunohistochemistry of native aortic valves showing morphology, high expression of Runx2 and osteopontin in the native aortic valves explanted 1, 32, and 53 months after TAVI. TBR indicates target-to-background ratio.

In 5 patients with TAVI for severe aortic stenosis, explanted TAVI valves and associated aortic roots were obtained 945 (range, 3–2044) days after implantation (Tables I and II in the Data Supplement). Calcified native aortic valve tissue was present around the perimeter of the TAVI bioprostheses (Figure 2) and histologically demonstrated evidence of ongoing calcification activity with increased staining for both osteopontin and Runx-2 (Figure 2, Figures I and II in the Data Supplement).

#### ^18^F-NaF Positron Emission Tomography

On contrast CT angiography at baseline, residual calcification from the native aortic valve was seen around the perimeter of the TAVI bioprosthesis in all cases. All subjects demonstrated ^18^F-NaF uptake surrounding the TAVI bioprostheses that originated from the native aortic valve tissue (TBR range, 1.6–5.8; Figure 2). Native valve ^18^F-NaF uptake was highest in patients imaged 5 years after TAVI (TBR, 3.3 [2.6–3.9] versus 2.2 [1.9–2.5] in those imaged 1 month after TAVI, *P*=0.023; Figure 2). Overall native valve uptake showed a modest positive correlation with the time from TAVI (*r*=0.36, *P*=0.023).

### Assessments of Bioprosthetic Valve Degeneration

#### Ex Vivo Validation

**Figure 3.**
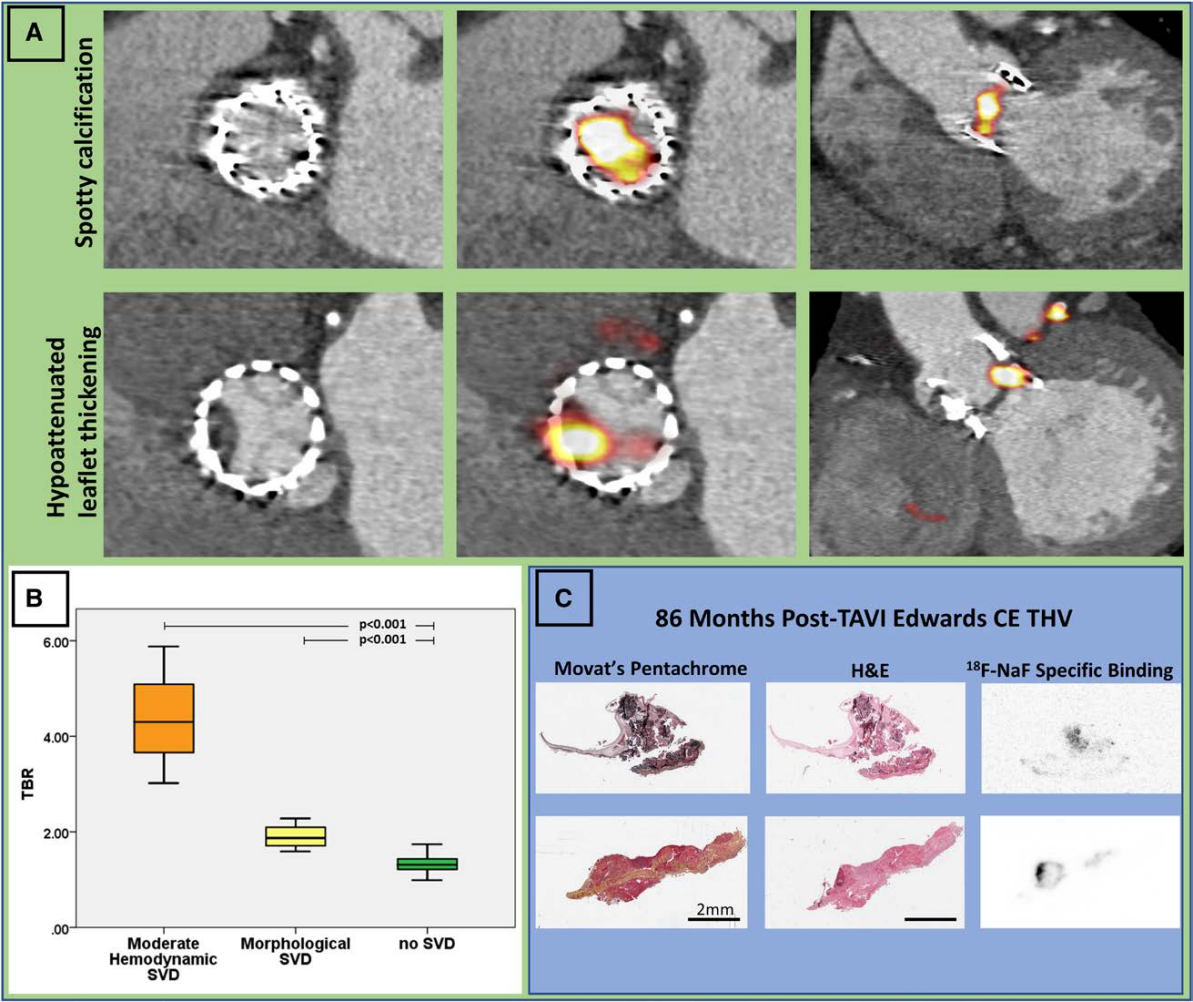
**^18^F-Sodium fluoride identifies early TAVI bioprosthetic valve degeneration.** **A**, **Top**, A 76-year-old woman with hemodynamic valve deterioration on echocardiography imaged 5 years after transcatheter aortic valve implantation (TAVI). Computed tomography angiography revealed spotty calcification on the bioprosthetic leaflets. On ^18^F-sodium fluoride (^18^F-NaF) positron emission tomography, we detected very high uptake in the leaflets (target-to-background [TBR]=5.9). The patient developed bioprosthesis failure 18 months after baseline positron emission tomography and underwent a successful TAVI-in-TAVI. **Middle**, An 88-year-old man with hemodynamic valve deterioration on echocardiography imaged 5 years after TAVI. Computed tomography angiography revealed hypoattenuated leaflet thickening. On ^18^F-NaF positron emission tomography we detected very high uptake in the leaflets (TBR=3.8). **B**, There was a stepwise increase in TAVI ^18^F-NaF uptake according to the presence and severity of valve dysfunction. ^18^F-NaF uptake was highest in patients with hemodynamic dysfunction, and more pronounced in those with structural valve deterioration (SVD) than normal TAVI valves. **C**, Histological and autoradiography validation of ^18^F-NaF avidity in an Edwards Conformitè Europëenne (CE) TAVI valve explanted after 86 months: Movat pentachrome and hematoxylin and eosin (H&E) staining demonstrate that leaflet calcification corresponds closely with ^18^F-NaF binding on autoradiography. THV indicates transcatheter heart valve.

In 4 explanted TAVI valves with evidence of valve leaflet degeneration, increased ^18^F-NaF uptake was seen on autoradiography, with colocalization of this signal to regions of calcification within the TAVI valve leaflets as observable on hematoxylin and eosin and Movat pentachrome staining (Figure 3).

#### Baseline Echocardiography and CT

On echocardiography during their baseline research visit, valve function was normal in all but 3 patients. These 3 patients had 5-year-old TAVI valves and demonstrated increased transvalvular gradients. This had not been appreciated on previous clinical echocardiograms or clinical follow-up. No patient had clinically significant valvular regurgitation. Leaflet morphology was assessable in 77% of patients, and no abnormalities were detected on baseline echocardiograms.

CT scans had image quality suitable for leaflet assessments in 87% of patients. Only 1 patient had evidence of TAVI leaflet calcification on CT, demonstrating spotty calcification that was just discernible from the valve struts (Figure 3). Pannus formation was not observed in any of our patients. HALT was found in 6 (13%) patients, 5 of whom were imaged 5 years after TAVI and 1 imaged 1 month after implantation. Four of these patients demonstrated minimal (<25%) leaflet involvement, whereas 2 patients had pronounced HALT (exceeding 50% of the leaflets) causing restricted single-leaflet motion on 4-dimensional CT. One patient with HALT had evidence of hemodynamic valve deterioration on echocardiography (mean pressure gradient, 24 mm Hg).

Overall, 8 patients had imaging evidence of bioprosthetic TAVI valve degeneration on echocardiography or CT. Seven of these patients were in the cohort of patients imaged 5 years after TAVI, with no differences in their baseline clinical characteristics compared with patients with similar-aged TAVI valves but normal imaging (Table III in the Data Supplement).

#### Baseline ^18^F-NaF PET

All patients had good image quality enabling the assessment of ^18^F-NaF uptake in the bioprosthetic leaflets. There was no difference in ^18^F-NaF uptake in self-expandable versus balloon-expandable TAVI bioprostheses (TBR, 1.3 [1.2–1.6] versus 1.3 [1.2–1.7]; *P*=0.74). We detected ^18^F-NaF uptake localized to the TAVI leaflets in 7 patients (15%), all imaged 5 years after TAVI (TBR range, 1.6–5.9). Valve TBR values were nearly double those in patients without visually apparent leaflet uptake (2.3 [1.7–4.3] versus 1.3 [1.2–1.4]; *P*<0.001). The 3 highest TBR values (range, 3.0–5.9) were observed in the patients with evidence of hemodynamic structural valve deterioration on echocardiography (stage 2 structural valve deterioration; mean transprosthetic pressure gradients >20 mm Hg). Increased uptake was also observed in patients with structural evidence of valve degeneration on CT (stage 1 structural valve deterioration) compared with valves with normal echocardiographic and CT appearances (Figure 2). One patient had evidence of increased ^18^F-NaF leaflet uptake in the absence of any changes on CT or echocardiography. Of 6 patients presenting with HALT, 4 showed increased ^18^F-NaF TAVI leaflet uptake (Figure 3 and Figure III in the Data Supplement).

#### Disease Progression and Clinical Outcomes

**Figure 4.**
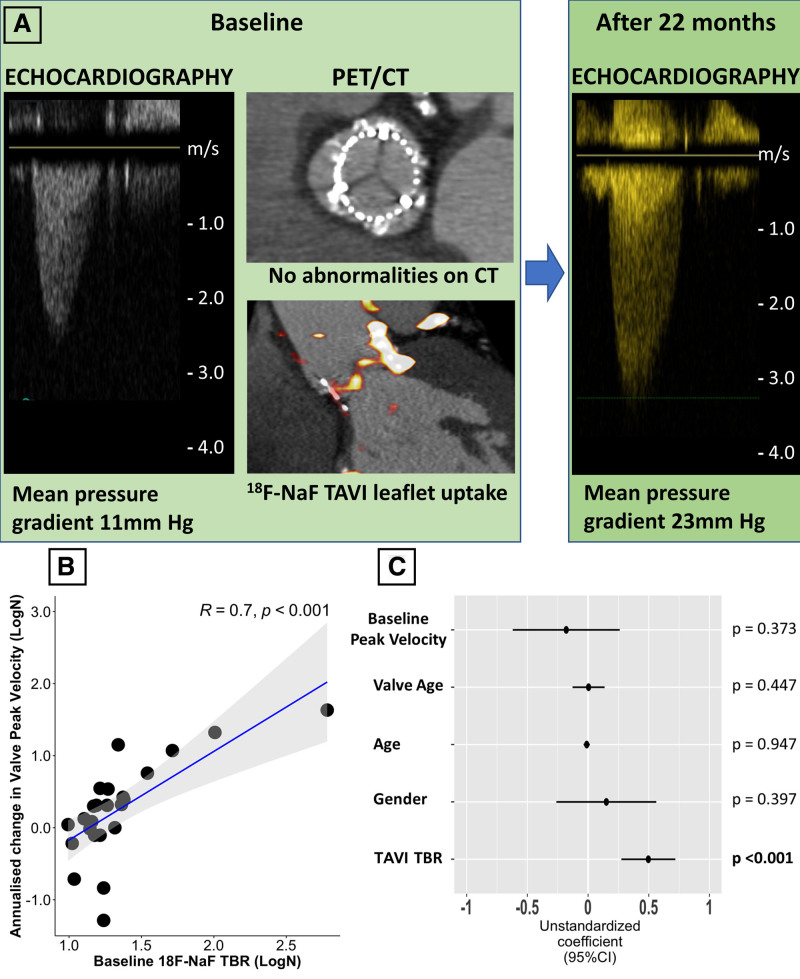
**Baseline ^18^F-sodium fluoride uptake predicts subsequent deterioration in TAVI function.** **A**, Case example of an 84-year-old patient imaged 5 years after transcatheter aortic valve implantation (TAVI). We detected TAVI ^18^F-sodium fluoride (^18^F-NaF) leaflet uptake in the absence of abnormalities on echocardiography (mean pressure gradient 11 mm Hg) and computed tomography (CT). At follow-up, the patient developed moderate bioprosthesis stenosis with a mean pressure gradient of 23 mm Hg. **B**, A strong correlation was observed between baseline ^18^F-NaF uptake in the TAVI valves (TBR) and subsequent progression in bioprosthetic valve peak velocity (*r*=0.7; *P*<0.001). **C**, Forest plot of unstandardized coefficients (95% CIs) from a multivariable linear regression analysis predicting change in TAVI valve function (annualized change in peak velocity) during follow-up. When examining all relevant baseline characteristics, ^18^F-NaF uptake was the only independent predictor of hemodynamic TAVI deterioration. PET indicates positron emission tomography; and TBR, target-to-background ratio.

Patients with TAVI underwent repeat echocardiographic evaluation at 15 (12–17) months to assess for evidence of progressive valve dysfunction. A strong correlation was observed between baseline ^18^F-NaF TBR values in the TAVI leaflets and the subsequent annualized change in bioprosthetic valve peak velocity on echocardiography (*r*=0.70, *P*<0.001; Figure 4). Similar correlations were observed between ^18^F-NaF leaflet uptake and the change in the mean pressure gradient (*r*=0.55, *P*=0.01) and the change in the effective orifice area (*r*=–0.71, *P*=0.007). On univariable analysis, the only predictors of the annualized change in peak velocity were valve age (*P*=0.035), abnormal CT findings (*P*=0.006), and ^18^F-NaF leaflet uptake (*P*<0.001; Table 2). On multivariable analysis incorporating age, sex, duration of valve implantation, baseline peak prosthetic valve velocity, and abnormal CT findings, ^18^F-NaF uptake was the only predictor of the annualized change in peak velocity (*P*<0.001; Table 3).

**Table 2.**
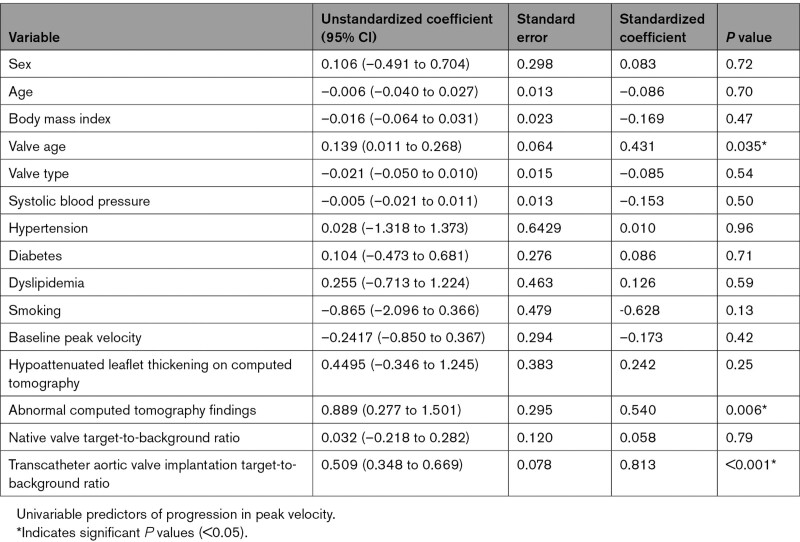
Factors Associated With Future Deterioration in Transcatheter Aortic Valve Implantation Function (Annualized Change in Peak Velocity After 2 Years): Univariable Analysis

**Table 3.**
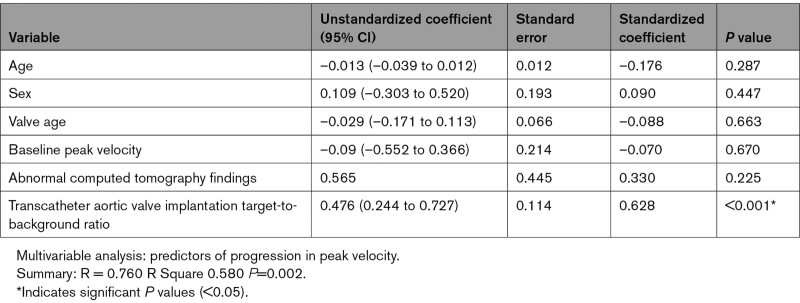
Factors Associated With Future Deterioration in Transcatheter Aortic Valve Implantation Function (Annualized Change in Peak Velocity After 2 Years): Multivariable Analysis

Four patients developed clinical criteria for hemodynamic structural valve deterioration during the follow-up period, with each developing bioprosthetic valve stenosis (mean pressure gradient, 27 [24–31] mm Hg and peak velocity 3.6 [3.4–4.1] m/s). Three patients had increased ^18^F-NaF TAVI leaflet uptake at baseline. In the single patient without increased ^18^F-NaF uptake at baseline, the increased mean pressure gradient normalized after 3 months of anticoagulation therapy and, in retrospect, was attributed to valve thrombosis rather than established irreversible structural valve disease. The patient with the highest leaflet ^18^F-NaF uptake in the TAVI cohort developed bioprosthesis failure 18 months after baseline PET and underwent a successful TAVI-in-TAVI. Based on the Youden index, the optimal cutoff TBR value to identify patients at increased risk of structural valve degeneration was 1.59. In our study, the 1.59 TBR threshold had a sensitivity of 86%, specificity of 89%, positive predictive value of 86%, negative predictive value of 97%, and accuracy of 89% for prediction of hemodynamic valve degeneration.

#### Comparison With Patients Who Had Age-Matched SAVR Valves

Fifty-one patients with SAVR who underwent the same research imaging protocol were compared with the 47 patients with TAVI. The latter were older (82 [76–86] versus 72 [70–77] years; *P*<0.001) and had more comorbidity than patients with SAVR. The time from valve replacement to imaging was similar (24 [24–60] versus 24 [24–60] months; *P*=0.91) as were the number of patients with SAVR and TAVI imaged 1 month, 2 years, and 5 years after valve replacement (Table 1). Patients with TAVI had lower peak aortic jet velocity (2.4 [2.0–2.7] versus 2.7 [2.4–3.0] m/s; *P*=0.03) and larger effective orifice area (1.5 [1.3–1.8] versus 1.1 [1.0–1.5] cm^2^; *P*=0.02; Table 1) than patients with SAVR.

**Figure 5.**
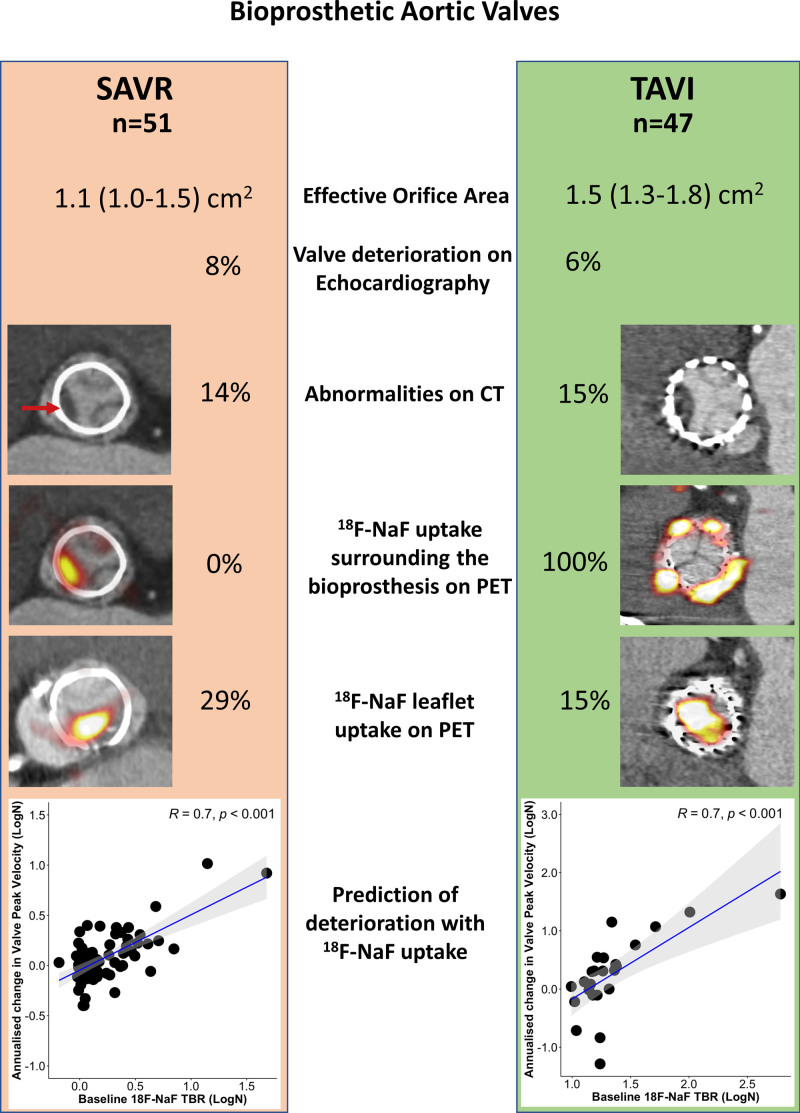
**Comparison of imaging findings and valve deterioration in TAVI vs bioprosthetic SAVR.** We compared echocardiographic, computed tomography (CT) and ^18^F-sodium fluoride (^18^F-NaF) findings in 47 patients with transcatheter aortic valve implantation (TAVI) with 51 patients with surgical aortic valve replacement (SAVR) who underwent the same research imaging protocol. We observed ^18^F-NaF uptake on the peripheral of all TAVI valves and none of the SAVR valves. Although patients with TAVI showed lower peak velocity (2.4 [2.0–2.7] vs 2.7 [2.4–3.0] m/s; *P*=0.03) and larger effective orifice area (1.5 [1.3–1.8] vs 1.1 [1.0–1.5] cm^2^; *P*=0.02) than patients with SAVR, we detected baseline echocardiographic (6% vs 8%; *P*=0.78) and CT abnormalities (15% vs 14%; *P*=0.87) suggestive of bioprosthetic degeneration in a similar proportion of patients with either TAVI or SAVR. The overall prevalence of patients with increased leaflet ^18^F-NaF uptake was nearly double in patients with SAVR compared with those with TAVI (29% and 15%; *P*=0.09). In both patients with SAVR or TAVI, baseline ^18^F-NaF leaflet uptake was predictive of the change in the peak transvalvular velocity on echocardiography. PET indicates positron emission tomography; and TBR, target-to-background ratio.

Evidence of bioprosthetic degeneration was similar in TAVI and SAVR groups on echocardiography (6% versus 8%, respectively; *P*=0.78) and CT (15% versus 14%, respectively; *P*=0.87; Figure 5). Although the overall prevalence of patients with increased leaflet ^18^F-NaF uptake appeared to be nearly double in patients with SAVR (29% versus 15% in those with TAVI), this did not reach statistical significance (*P*=0.09), and, in those studied at 5 years, there was no difference in the proportion of patients demonstrating bioprosthetic uptake (40% patients with SAVR versus 44% patients with TAVI; *P*=0.79). Overall ^18^F-NaF uptake was similar in both TAVI and SAVR valves (TBR: 1.3 [1.2–1.7] versus 1.3 [1.2–1.5]; *P*=0.27).

## Discussion

In patients with TAVI, we have demonstrated that ^18^F-NaF uptake within the native aortic valve is higher with longer duration of implantation, suggesting that disease activity continues despite immobilization of the valve leaflet. This was further supported by our histological finding of continued activation of procalcific markers in explanted native valves after TAVI. We have further shown by using 3 complementary and distinct imaging modalities that the prevalence of valve degeneration within TAVI bioprostheses is similar to that of bioprosthetic SAVR valves for up to 7 years after valve replacement. Last, we have confirmed that ^18^F-NaF PET of the bioprosthetic valve provides a powerful independent predictor of subsequent hemodynamic bioprosthetic valve degeneration that is applicable to both TAVI and SAVR and outperforms all other traditional risk factors. We conclude that aortic stenosis is an active regulated disease process rather than solely the result of simple wear and tear of the valve, and that TAVI appears to have durability similar to SAVR with comparable modest rates of midterm bioprosthetic valve degeneration.

We have previously established ^18^F-NaF PET as a tool for the in vivo assessment of calcification activity across multiple different cardiovascular disease states.^10–15^ In patients with aortic stenosis, valvular ^18^F-NaF uptake provides an assessment of disease activity and prediction of subsequent disease progression and clinical events.^10,11^ We have here demonstrated that ^18^F-NaF uptake continues to occur in the retained native aortic valve of all patients with TAVI. We had hypothesized that ^18^F-NaF uptake might have transiently increased early after TAVI when native valve calcium has been disrupted, thereby increasing the available surface area for ^18^F-NaF binding. Thereafter, ^18^F-NaF uptake would be anticipated to decline as the valve heals and the mechanical trauma of repeated valve closure ceased. However, we observed the opposite. Native aortic valve ^18^F-NaF uptake and calcification activity was higher with longer duration of implantation. We observed a modest correlation between native valve uptake and the time from TAVI. This finding was supported by our ex vivo data that demonstrated histological evidence of ongoing calcification activity in native aortic valve tissue many years after TAVI. These observations are consistent with the hypothesis that, once established, calcification activity in the native aortic valve continues to accelerate in an ongoing pathobiological process with continuing mineralization (the propagation phase) that is not halted even after TAVI.^28^ The fact that it continues several years after TAVI, when mechanical stresses are no longer being exerted on the valve leaflets, confirms that aortic stenosis is an active regulated disease process and not simply the result of valve wear and tear. Therapies focused on slowing this cycle of calcification are required if we are going to develop the medical treatments for aortic stenosis that are so urgently needed. Medications interfering with tissue calcification and ectopic bone formation (alendronate and denosumab) have recently been tested in this context but unfortunately were unable to alter aortic valve calcification or disease progression.^5,29,30^

In patients with bioprosthetic SAVR, ^18^F-NaF uptake provides a marker of bioprosthetic valve degeneration and a powerful predictor of subsequent valve dysfunction.^12^ Our present study extends these findings to patients with TAVI, demonstrating that increased ^18^F-NaF uptake in the bioprosthetic valve leaflets provides an early indication of valve degeneration and a more powerful predictor of subsequent valve dysfunction than valve age, cardiovascular comorbidities, and imaging assessments provided by echocardiography and CT. The association between baseline bioprosthetic leaflet ^18^F-NaF uptake and subsequent change in bioprosthetic valve peak velocity was identical in patients with TAVI (*r*=0.7, *P*<0.001) to that previously reported for bioprosthetic SAVR valves (*r*=0.7, *P*<0.001). Combined with the existing bioprosthetic SAVR data, this positions ^18^F-NaF PET as a highly promising marker of early bioprosthetic valve degeneration that might provide important value in the prediction of bioprosthesis failure, in particular, because other imaging modalities such as echocardiography and CT are currently limited in this regard. Future trials are now required to assess whether this molecular imaging technique can aid clinical decision-making and risk stratify patients with bioprosthetic valves. Based on the findings of this study, 1 potential strategy would be to perform a 5-year ^18^F-NaF PET scan after TAVI as a screening tool for identifying those at increased risk of rapid deterioration. This might help the planning of repeat intervention and differentiate patients who require close monitoring from those with no evidence of even early valve degeneration who can be assessed much less frequently.

Given the powerful prediction of valve dysfunction provided by ^18^F-NaF in both bioprosthetic SAVR and TAVI valves, our data set provides a unique opportunity to compare early valve degeneration in age-matched bioprosthetic SAVR and TAVI valves, thereby helping address one of the most important current questions in heart valve disease. Are TAVI valves likely to last as long as surgical bioprostheses? In the present study, there were no differences in the proportion of patients with TAVI or SAVR bioprostheses who had echocardiographic or CT evidence of valve degeneration for up to 7 years after replacement. Very similar rates of increased ^18^F-NaF uptake were observed in patients with SAVR and TAVI valves implanted 5 years previously (40% versus 44%) despite patients with TAVI having a much higher burden of cardiovascular comorbidities. Taken together, our data suggest that imaging assessments of valve degeneration are similar between these 2 types of valve, supporting similar midterm durability of TAVI and SAVR bioprosthetic valves. If confirmed in larger studies, then this would help assuage one of the main lingering concerns about performing TAVI as the first-line valve replacement method in patients with aortic stenosis.

Our study has several strengths and weaknesses. We have used a state-of-the-art multimodality imaging study design and used the same protocols to image patients with age-matched SAVR and TAVI valves, thereby providing a unique opportunity to compare imaging findings in these 2 valve types. Moreover, we provide longitudinal data confirming the predictive value of ^18^F-NaF PET in both SAVR and TAVI valves. Although relatively large for a complex molecular imaging study, our overall sample size is modest (47 TAVI and 51 SAVR valves). Our observations therefore require confirmation in larger data sets with longer follow-up. Patients with bioprosthetic SAVR and TAVI were not matched for age or comorbidities; however, given the different patient populations who currently have received these 2 treatments, this is inevitable, and our results would suggest that these comorbidities do not greatly influence valve degeneration or durability. Given the cross-sectional nature of our study, we acknowledge the potential for survivor bias. This could be addressed in future longitudinal cohort studies to ensure prospective capture of all cases of valvular degeneration. As a result of the outbreak of SARS-CoV-2 pandemic, we discontinued further recruitment before reaching our predefined number of study participants; therefore, further studies are needed to confirm our findings. Last, in our study, we focused on bioprosthetic valves, and our findings should not be extrapolated to mechanical aortic valve prostheses that have better durability than both forms of bioprosthetic valve.

In conclusion, we have demonstrated that native aortic valves after TAVI demonstrate evidence of ongoing disease activity, suggesting that aortic stenosis is an active disease process that is independent of motion and mechanical injury. Across 3 complementary and distinct imaging modalities, TAVI degeneration appears to be of a similar magnitude to bioprosthetic SAVR, suggesting comparable midterm durability. ^18^F-NaF PET appears to be a consistent method of detecting early bioprosthetic valve degeneration and predicting subsequent dysfunction for both TAVI and SAVR.

## Sources of Funding

This research was supported in part by grant R01HL135557 from the National Heart, Lung, and Blood Institute/National Institute of Health (NHLBI/NIH). The content is solely the responsibility of the authors and does not necessarily represent the official views of the National Institutes of Health. Drs Newby (CH/09/002, RE/18/5/34216, RG/16/10/32375), Tzolos (FS/CRTF/20/24086), Fletcher (FS/19/15/34155), Gulsin (FS/TF/21/33008), Williams (FS/ICRF/20/26002), Barton (SS/CH/09/002/26360), and Dweck (FS/14/78/31020) are supported by the British Heart Foundation. Dr Tzolos was supported by a grant from Dr. Miriam and Sheldon G. Adelson Medical Research Foundation. Dr Newby is the recipient of a Wellcome Trust Senior Investigator Award (WT103782AIA) and Dr Dweck is the recipient of the Sir Jules Thorn Award for Biomedical Research Award (2015). Dr Sellers is supported by fellowships from the Michael Smith Foundation for Health Research and the Canadian Institutes of Health Research. Dr van Beek is supported by Scottish Imaging Network (www.sinapse.ac.uk). Dr Dey is supported by NHLBI/NIH grants 1R01HL148787-01A1 and 1R01HL151266-01. Dr Rudd is supported in part by the National Institute for Health Research Cambridge Biomedical Research Center, the British Heart Foundation, Higher Education Funding Council for England, the Engineering and Physical Sciences Research Council, and the Wellcome Trust. Dr Tarkin is supported by a Wellcome Trust Clinical Research Career Development Fellowship (211100/Z/18/Z). Dr Leipsic is supported by a Canadian Research Chair in Advanced CardioPulmonary Imaging and the Jon Dehaan Award for Innovation in Cardiology.

## Disclosures

Dr Leipsic consults for Heartflow Inc and Circle Cardiovascular Imaging and provides computed tomography core laboratory services for Edwards Lifesciences, Medtronic, Boston Scientific, Abbott, PI Cardia, MVRX, for which no direct compensation is received. Dr Leipsic has stock options in HeartFlow Inc and Circl CVI. Drs Cruden and Uren proctor for Edward Lifesciences. The other authors report no conflicts.

## Supplemental Materials

Expanded Methods

Data Supplemental Tables I–III

Data Supplemental Figures I–III

## Supplementary Material


