# Supplementary material for: Native Aortic Valve Disease Progression and Bioprosthetic Valve Degeneration in Patients With Transcatheter Aortic Valve Implantation
Source: Circulation. 2021 Aug 29;144(17):1396–408. doi: 10.1161/CIRCULATIONAHA.121.056891 (PMC8542078; doi:10.1161/CIRCULATIONAHA.121.056891)

Native aortic valve disease progression and bioprosthetic valve degeneration in patients with transcatheter aortic valve implantation

Jacek Kwiecinski MD, PhD[1*], Evangelos Tzolos MD[2*], Timothy RG Cartlidge MD, PhD[2*], Alexander Fletcher MD[2], Mhairi K Doris MD, PhD[2], Rong Bing MD[2], Jason M Tarkin MD, PhD[6], Michael A Seidman MD[3], Gaurav S. Gulsin MD[3], Nicholas L Cruden MD[2], Anna K Barton MD[2], Neal G Uren MD[2], Michelle C Williams MD, PhD[2], Edwin JR van Beek MD, PhD[2,4], Jonathon Leipsic MD[3], Damini Dey PhD[5], Raj R Makkar MD[5], Piotr J Slomka PhD[5], James HF Rudd MD, PhD[6], David E Newby MD, PhD[2], Stephanie L Sellers MSc, PhD[3#], Daniel S Berman MD[5#], Marc R Dweck MD, PhD[2#]

Supplemental Material

Expanded Methods

Ex Vivo Analysis

To assess calcification activity in native aortic valves, tissue samples were selected from TAVI valves that had been harvested *en bloc* along with the aortic root and the surrounding native valve tissue. Immunohistochemistry was then performed on all native aortic valve explants: 4-µm paraffin sections of the native valve tissue were cut and stained for Runx2 and Osteopontin. Dilutions of 1:100 and 1:200 were used for Runx2 (Abcam, Ab76956) and osteopontin (Sigma-Aldrick Cat. No. 07264) respectively. For both Runx2 and osteopontin, staining were performed using the automated Leica Bond Rx system, Bond Epitope Retrieval Solution 1 (pH=6, Catalog No: AR9961) and Bond Polymer Refine Red Detection (Catalog No: DS9390).

To assess whether ^{18}F-NaF is providing an assessment of calcification activity in TAVI valve leaflets, TAVI valves of different implant durations were selected for evaluation. Hematoxylin and eosin (H&E) and Movat's pentachrome staining was performed on 4um paraffin sections of TAVI leaflets. Slides were imaged on a high-resolution Aperio Slide Scanner with images generated using ImageScope software (Leica Biosystems, Germany). For ^{18}F-NaF autoradiography experiments, slide-mounted paraffin-

embedded sections were rehydrated bathed in PBS and placed in 100 KBq/mL of ^{18}F-NaF for 1 hour, before being washed in PBS again. The slides were then placed in the phosphor-images Amesham Typhoon (GE) at 4000 X 10 µm scale, the Fuji film high resolution film applied and left overnight for exposure. Autoradiography analysis was performed in the open-source software ImageJ (v2.0.0).

Positron Emission Tomography

Motion Correction

Motion correction and image analysis were performed using FusionQuant software (Cedars Sinai Medical Center, Los Angeles, CA; 23). Cardiac motion was corrected with an anatomically guided automated registration algorithm as described previously (25). First, a 3-dimensional sphere was drawn to define the aortic valve region. A nonlinear registration algorithm, radially constrained around the valve, was used to align PET images to the diastolic gate. The nonlinear registration algorithm was a diffeomorphic, mass-preserving, anatomy-guided demon method that optimizes the global energy between PET frames, with built-in optimization for anatomic data (26). The motion-corrected gates were then summed to form a motion-free image (26) containing all the PET counts.

Supplemental Table I: Post transcatheter aortic valve implantation native valve explant cases

	Case 1	Case 2	Case 3	Case 4	Case 5
Time to Explant, months	52	81	1	31	2
Age at death, years	72	96	76	99	88
Sex, (0=female, 1=male)	-	+	+	-	-
Medical history, (0=no, 1=yes)					
Hypertension	-	-	+	+	+
Coronary artery disease	-	+	+	-	+
Coronary bypass surgery	-	+	+	-	-
Diabetes	-	+	-	-	-
Hypercholesterolemia	-	+	+	-	+
Smoking history	-	+	+	-	+
Medication, (0=no, 1=yes)					
Aspirin	-	+	+	+	+
Clopidogrel	-	+	-	-	-
Warfarin	-	-	-	-	-
Other anticoagulant	+	-	-	-	-
ACE inhibitor or Angiotensin receptor blocker	+	+	+	+	+
Beta-blocker	-	+	+	-	+
Statin	-	+	+	-	+
Pre-TAVI echocardiography					
LV ejection fraction, %	65	65	35	50	60
Mean valve gradient, mmHg	96	90	19*	76	24
Valve area, cm^2	0.47	0.48	0.8	0.4	0.7

* Mean gradient increased to 31 mmHg with dobutamine stress echo

ACE: angiotensin converting enzyme; LV: left ventricle; TAVI: transcatheter aortic valve implantation.

Supplemental Table II: Post transcatheter aortic valve implantation native valve explant cases demographics summary

	**n=5**
TAVI implant age (years)	31 [2-52]
Age at death (years)	88 [76-96]
Men	2 (40%)
Medical history	
Hypertension	3 (60%)
Coronary artery disease	3 (60%)
Coronary bypass surgery	2 (40%)
Diabetes	1 (20%)
Hypercholesterolemia	3 (60%)
Ex-smoker	3 (60%)
Medication	
Aspirin	4 (80%)
Clopidogrel	1 (20%)
Warfarin	0
Other anticoagulant	1 (20%)
ACE inhibitor or angiotensin receptor blocker	5 (100%)
Beta-blocker	3 (60%)
Statin	3 (60%)
Pre-TAVI echocardiography	
LV ejection fraction (%)	55 [50-60]
Mean valve gradient (mmHg)	76 [31-90]
Valve area (cm^2)	0.57 [0.47-0.70]

Number (%); median [interquartile range]

ACE - angiotensin converting enzyme; LV - left ventricle; TAVI - transcatheter aortic valve implantation.

Supplemental Table III. Comparison of patients with and without ^{18}F-sodium fluoride transcatheter aortic valve leaflet uptake.

	Patients with ^{18}F-NaF transcatheter aortic valve leaflet uptake (n=7)	Patients without ^{18}F-NaF transcatheter aortic valve leaflet uptake (n=40)	**P value**
Age (years)	81 [75-86)	82 [76-87)	0.68
Men	5 (63%)	24 (62%)	0.74
Body-mass index (kg/m^2)	25 [21-27]	24 [20-26]	0.47
Systolic blood pressure (mmHg)	130 [118-137)	132 [120-146]	0.72
Diastolic blood pressure (mmHg)	62 [59-70)	68 [60-74]	0.56
**Bioprosthesis age**			
time since valve implantation (months)	59 [59-60]	24 [24-24]	<0.001
5 years post valve implantation	7 (100%)	9 (23%)	<0.001
2 years post valve implantation	0	22 (55%)	<0.001
1 month post valve	0	8 (20%)	<0.001
**Comorbidities/risk factors, n (%)**			
Hypertension	6 (86%)	32 (80%)	0.63
Hyperlipidemia	6 (86%)	20 (50%)	0.40
Diabetes	2 (29%)	12 (30%)	0.54
Smoking	5 (71%)	23 (58%)	0.53
Coronary artery disease	4 (57%)	20 (50%)	N/A
Coronary artery bypass grafts	2 (29%)	15 (38%)	0.92
**Medications**			
Aspirin	4 (57%)	23 (58%)	0.39
P2Y12 antagonist	2 (29%)	6 (15%)	0.19

Warfarin	2 (29%)	5 (13%)	0.14
Direct oral anticoagulation	1 (14%)	0	N/A
ACE inhibitor/angiotensin receptor blocker	5 (71%)	25 (63%)	0.68
Beta blocker	5 (71%)	23 (58%)	0.52
Statin	6 (86%)	29 (73%)	0.71
**Echocardiography**			
Peak Valve Velocity (m/s)	2.9 [2.3-3.4]	2.0 [1.7-2.3]	<0.001
Effective orifice area (cm^2)	1.0 [0.8-1.3]	1.8 [1.6-2.2]	<0.001
Mean valve gradient (mmHg)	19 [17-22]	12 [10-15]	<0.001
LV ejection fraction (%)	60 [55-60]	58 [55-60]	0.46
**Electrocardiogram**			
Sinus rhythm	5 (71%)	22 (55%)	0.58
Paced rhythm	2 (29%)	7 (18%)	N/A
Atrial fibrillation	3 (43%)	4 (10%)	N/A
LV hypertrophy	1 (14%)	4 (10%)	0.35
Strain pattern	0	3 (8%)	N/A
**^{18}F-NaF uptake**			
Bioprosthetic uptake	7 (100%)	0	N/A
Target to background ratio	2.3 [1.7-4.3]	1.3 [1.1-1.5]	<0.001

Number (%); median [interquartile range]

ACE – angiotensin-converting enzyme; ^{18}F-NaF - ^{18}F-sodium fluoride

Supplemental Figure I. Further examples of native aortic valve ^{18}F-sodium fluoride uptake following transcatheter aortic valve implantation.

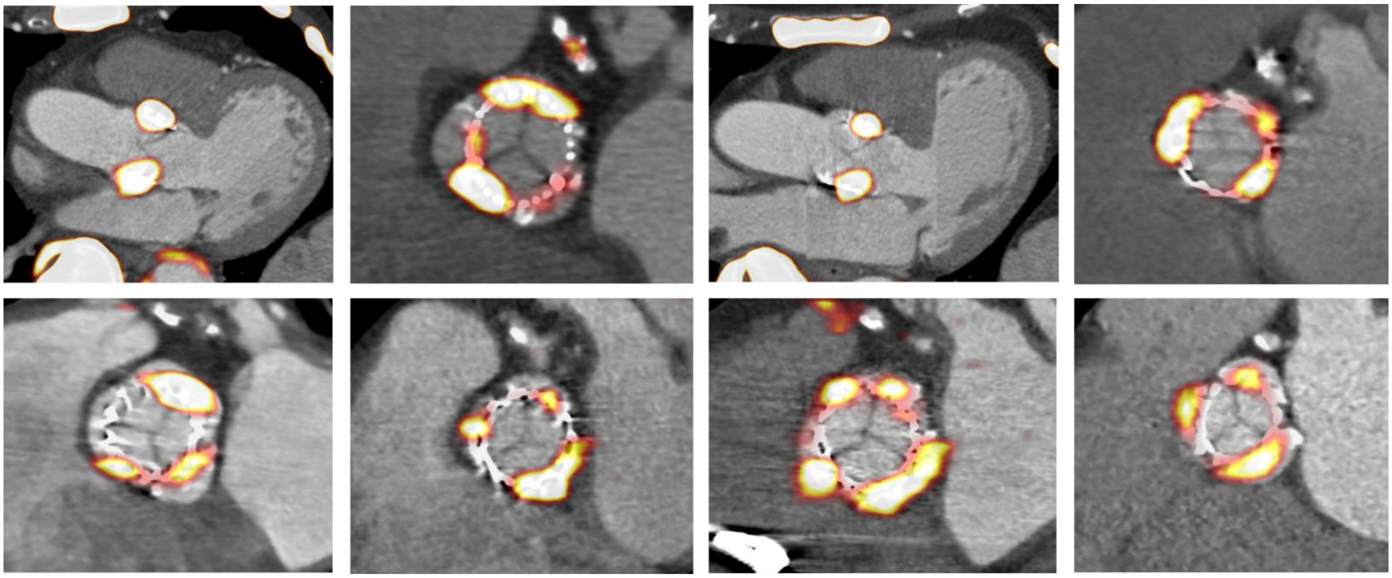

Supplemental Figure II: Histology of native aortic valves explanted post-transcatheter aortic valve implantation. Representative images of histological sections with Movat's pentachrome staining of left, right and non-coronary cusps (LCC, RCC, NCC) showing severely calcified valves at time of post-mortem explant in patients having undergone transcatheter aortic valve implantation. Histological images are shown at 1.7x.

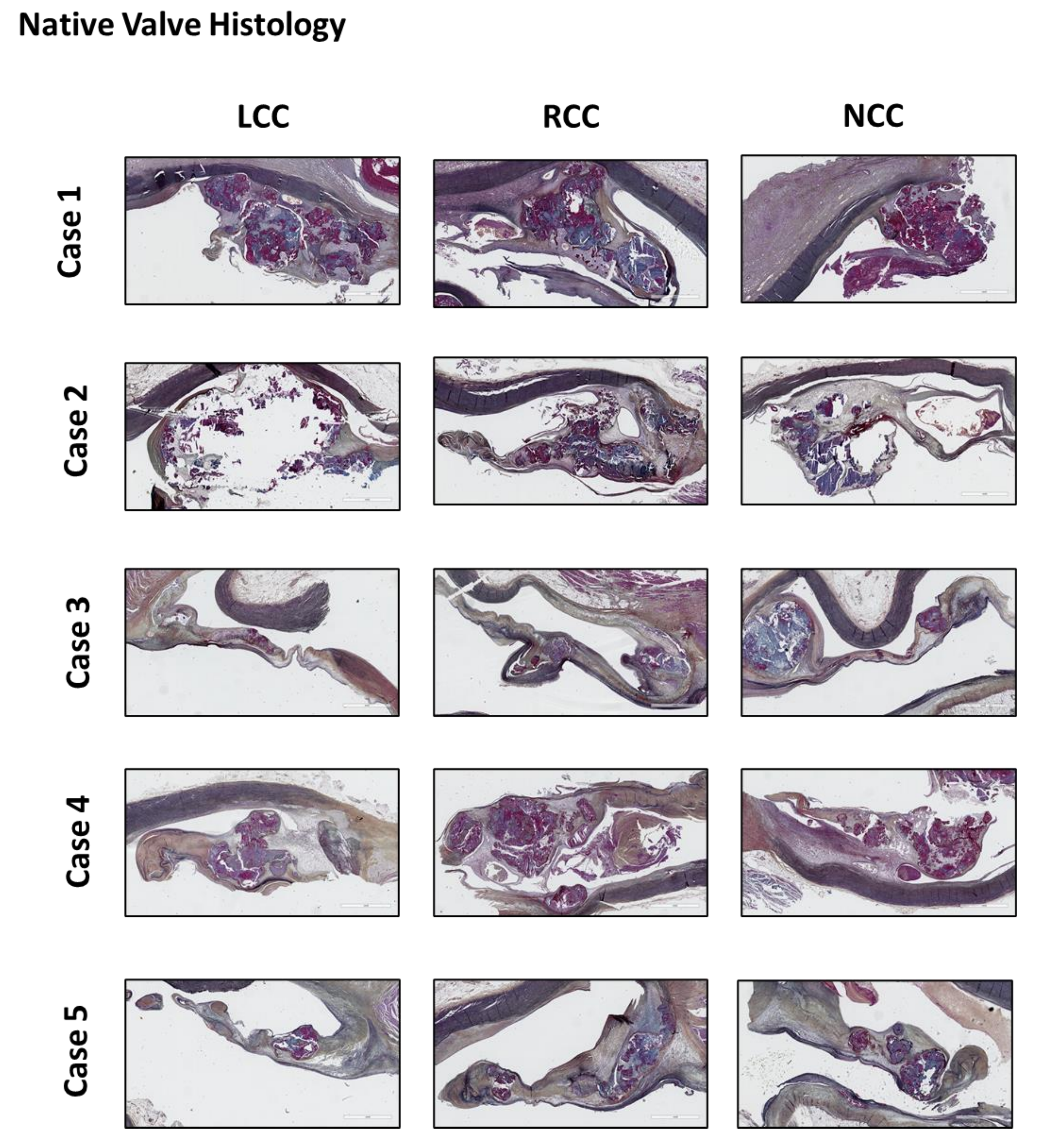

LCC - left coronary cusp; RCC - right coronary cusp; NCC - non-coronary cusp

Supplemental Figure III. Further examples of increased ^{18}F-sodium fluoride uptake in the leaflets of transcatheter bioprosthesis.

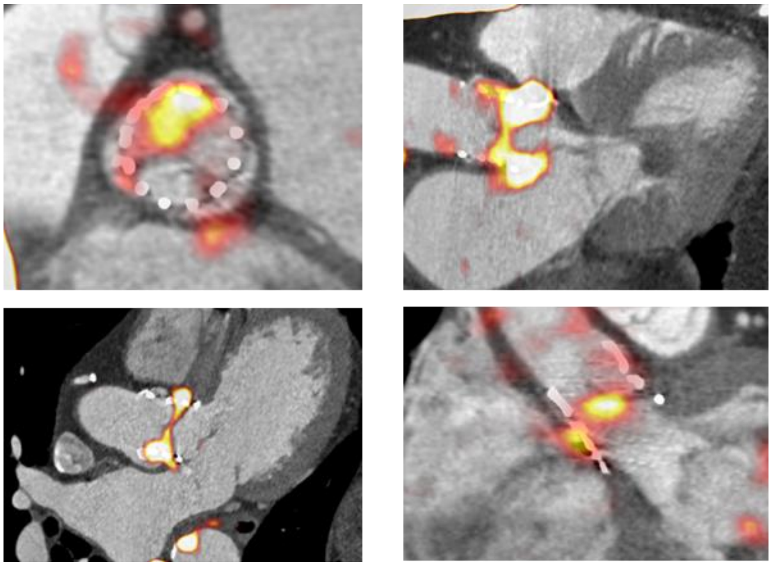

Supplement: Supplementary file 1 [file cir-144-1396-s001.pdf]
